# Why location matters: associations between county-level characteristics and availability of National Cancer Oncology Research Program and National Cancer Institute sites

**DOI:** 10.1093/jncics/pkae038

**Published:** 2024-05-14

**Authors:** Nicole E Caston, Courtney P Williams, Emily B Levitan, Russell Griffin, Andres Azuero, Stephanie B Wheeler, Gabrielle B Rocque

**Affiliations:** Division of Hematology and Oncology, Department of Medicine, University of Alabama at Birmingham (UAB), Birmingham, AL, USA; Division of Preventive Medicine, University of Alabama at Birmingham Department of Medicine, Birmingham, AL, USA; O’Neal Comprehensive Cancer Center, University of Alabama at Birmingham, Birmingham, AL, USA; Department of Epidemiology, University of Alabama at Birmingham School of Public Health, Birmingham, AL, USA; Department of Epidemiology, University of Alabama at Birmingham School of Public Health, Birmingham, AL, USA; University of Alabama at Birmingham School of Nursing, Birmingham, AL, USA; Department of Health Policy and Management, Gillings School of Public Health, University of North Carolina (UNC), Chapel Hill, NC, USA; University of North Carolina Lineberger Comprehensive Cancer Center, Chapel Hill, NC, USA; Division of Hematology and Oncology, Department of Medicine, University of Alabama at Birmingham (UAB), Birmingham, AL, USA; O’Neal Comprehensive Cancer Center, University of Alabama at Birmingham, Birmingham, AL, USA

## Abstract

**Background:**

The majority of patients with cancer seek care at community oncology sites; however, most clinical trials are available at National Cancer Institute (NCI)–designated sites. Although the NCI National Cancer Oncology Research Program (NCORP) was designed to address this problem, little is known about the county-level characteristics of NCORP site locations.

**Methods:**

This cross-sectional analysis determined the association between availability of NCORP or NCI sites and county-level characteristic theme percentile scores from the Center for Disease Control and Prevention’s Social Vulnerability Index themes. Health Resources and Services Administration’s Area Health Resource Files were used to determine contiguous counties. We estimated risk ratios and 95% confidence intervals (CIs) using modified Poisson regression models to evaluate the association between county-level characteristics and site availability within singular and singular and contiguous counties.

**Results:**

Of 3141 included counties, 14% had an NCORP, 2% had an NCI, and 1% had both sites. Among singular counties, for a standard deviation increase in the racial and ethnic theme score, there was a 22% higher likelihood of NCORP site availability (95% CI = 1.10 to 1.36); for a standard deviation increase in the socioeconomic status theme score, there was a 24% lower likelihood of NCORP site availability (95% CI = 0.67 to 0.87). Associations were of smaller magnitude when including contiguous counties. NCI sites were located in more vulnerable counties.

**Conclusions:**

NCORP sites were more often in racially diverse counties and less often in socioeconomically vulnerable counties. Research is needed to understand how clinical trial representation will increase if NCORP sites strategically increase their locations in more vulnerable counties.

Though the majority of patients with cancer opt to receive cancer care at community oncology practices, recent estimates have suggested only 4% of the cancer patient population participated in clinical trials held at Community Sites compared with approximately 20% of patients who participated in trials held at National Cancer Institute (NCI)–designated sites (1-3). NCI-designated sites more often have the logistical resources, monetary means, and staff members to assist in conducting clinical trials. As health and well-being benefits are associated with clinical trial participation (eg, opportunity to receive a new treatment, access to less expensive treatment, increased interactions with providers, and the opportunity to be monitored more closely) (4-6), increased capacity to offer cancer clinical trials is needed to reach all patients with cancer.

The NCI National Cancer Oncology Research Program (NCORP) was designed to address inequities between where patients receive cancer care and where cancer clinical trials are offered. The mission of NCORP is to bring clinical trials to individuals in their own communities (7). NCORP sites are located across the United States (including Guam and Puerto Rico) with 7 Research Bases, 46 Community Sites (14 of those being Minority/Underserved Sites), and 1029 participating hospitals (which may include multiple clinics within 1 overarching hospital system). Hospitals participating in the NCORP network have access to the above-mentioned resources necessary to successfully conduct clinical trials. Furthermore, the Minority/Underserved Sites serve communities with a higher proportion of racially marginalized individuals. NCORP sites complement the 63 NCI-designated cancer centers within the United States where the majority of clinical trials are conducted (8).

Despite goals of serving populations with more barriers to clinical trial participation, NCORP sites reported 79% of the population they serve is White, 13% Black, 2.5% Asian, and 0.6% Native Hawaiian or Pacific Islander individuals. Additionally, 10% of patients identified as Hispanic (9). Little information is available comparing the social determinants of health of patients treated at NCORP and NCI centers compared with other settings. This calls into question whether key populations are reached by NCORP sites. For example, patients who live in rural (who are more often White individuals) (10) or disadvantaged areas (who are more often of marginalized racial and ethnic groups) (11,12) face transportation issues (eg, vehicle access, fuel or gas affordability, distance) which limits access to hospitals not in their community thus limiting access to novel treatment and technologies (13). Furthermore, because of a host of historical and current policies and practices, patients who are of historically marginalized racial groups participate in clinical trials less than their White counterparts (with as few as 3%-5% of Black patients participating in clinical trials) (14-19). One postulated approach for increasing clinical trial participation for patients in these areas is the use of telehealth to mitigate barriers related to distance. The pandemic-related increase in telemedicine usage within the US health-care system (20) has potential to extend the reach of NCORP, but internet access would be an important facilitator to patients participating in cancer clinical trials from community settings.

According to Levesque’s Conceptual Framework of Access to Health, geographical location, socioeconomic capital, and one’s living environments affect a person’s ability to reach, access, and utilize health care, which ultimately affects individual health (21). Questions remain about how area-level social vulnerability, including environmental, societal, logistical, and financial factors, may facilitate individuals’ ability to access NCORP and NCI sites; therefore, this study explores associations between county-level characteristics and availability of NCORP and NCI sites. Additionally, we explored the availability of internet as a marker of the potential for extended reach via telehealth.

## Methods

### Study design and setting

This cross-sectional study explored the association between county-level characteristics and availability of NCORP and NCI sites. NCORP and NCI address information was downloaded from the internet in October 2022 from the NCORP and NCI websites (7,22). Inclusion criteria included counties within the 50 states and the District of Columbia. This study was exempt from Institutional Review Board approval.

### Primary outcome: availability of NCORP and NCI sites

Individual counties were considered having access to an NCORP or NCI site if in the corresponding county (yes, no). A secondary outcome was inclusion of an NCORP or NCI site for the corresponding county and its contiguous counties (yes, no), as we hypothesized that the majority of patients would travel up to 1 county distance away to receive care. Additionally, the choice to use a binary outcome was influenced by the rarity of counties having more than 1 site. NCORP site locations were obtained from the NCORP website (7). NCI sites were obtained from the NCI site county ShapeFile dataset (22). Information on contiguous counties was available from the 2021-2022 Health Resources and Services Administration’s Area Health Resource Files (AHRF) (23). AHRF files contain county-level information for all US counties, including population, health care, economic, and environmental characteristics. For the purposes of this manuscript, we only included the following data from AHRF: contiguous county identifiers and Rural-Urban Continuum Codes (RUCCs).

### Secondary outcome: internet access

Additional information on the percentage of households with broadband internet was abstracted from the Centers for Disease Control and Prevention and Agency for Toxic Substances and Disease Registry’s Social Vulnerability Index (SVI) (24).

### Exposure: SVI

County-level characteristics were abstracted from the SVI dataset, which uses 2020 US Census data to determine community-level social vulnerability, defined as factors that could weaken a community’s ability to prevent human and financial loss following a disaster (24). A total of 16 variables make up 4 themes, and an overall vulnerability theme combines all 4 themes. All themes are percentile ranked from 0 to 1, with higher scores representing more vulnerability. Data were downloaded from the SVI website, and no data manipulation occurred.

#### Overall vulnerability theme

According to the Centers for Disease Control and Prevention and Agency for Toxic Substances and Disease Registry documentation, counties with scores 0.9-1.0 are considered the most vulnerable. We used this logic to dichotomize counties into high (≥90%) or low (<90%) vulnerability (24). The following variables are included for each theme.

*Socioeconomic status.* Percentage of individuals living below 150% poverty, unemployed, with no high school diploma, with no health insurance, and housing cost burden.

*Household characteristics.* Percentage of individuals aged 65 years and older, aged 17 years and younger, disabled, with English language proficiency, and single-parent households.

*Racial and ethnic minority status.* Percentage of individuals identifying as Hispanic or Latino (of any race); not Hispanic or Latino for Black and African American, American Indian and Alaska Native, Asian, Native Hawaiian and Other Pacific Islander, 2 or more races, other races.

*Housing type and transportation.* Percentage of multi-unit structures, mobile homes, crowding, group quarters, and individuals without a vehicle.

### Additional county-level variables

We included 2013 RUCC from the AHRF dataset for each county. RUCC scores range from 1 to 9 with scores 1-3 representing metropolitan counties, 4-6 suburban, and 7-9 rural (25,26). Furthermore, we included the 4 major US regions (Midwest, Northeast, South, and West) based on the corresponding state of the county. All datasets were merged by Federal Information Processing Standards (FIPS) codes at the county level.

### Statistical analysis

Descriptive statistics were calculated using frequencies and percentages for categorical variables and means and standard deviations (SD) for continuous variables. Differences in county-level characteristics were calculated using measures of effect size such as Cohen’s *d* (ie, the standardized mean difference; small: 0.2; medium: 0.5; large: 0.8) or Cramer’s V, which is based on the χ^2^ statistic. A Cramer’s V of 0.1 is considered a small effect, 0.3 a medium effect, and 0.5 a large effect when comparing across 2 categories and 0.1 a small effect, 0.25 a medium effect, and 0.4 a large effect when comparing across more than 2 categories (27). Risk ratios (RRs) and 95% confidence intervals (CIs) were estimated using modified Poisson regression models with robust standard errors evaluating the association between county-level characteristics and availability of an NCORP or NCI site. Because the data contain information on almost all US counties (ie, there is no sampling of counties), inferential quantities measuring sampling uncertainty such as standard errors and confidence intervals are applicable to different periods of time (ie, the current time period constitutes 1 random period taken from the population of cohort time periods). The first set of models assessed singular county access to sites, and the second set assessed county and its contiguous counties access to sites. For the first set of models (singular county), we tested the association between multiple specifications of the SVI variable as continuous and dichotomized and NCORP and NCI availability. For NCORP site availability, a third model was fitted containing, as predictors, all 4 SVI themes and RUCC. However, no singular rural counties had an NCI site; therefore, the singular county third model for NCI availability was fitted containing only the 4 SVI themes. For the second set of models (contiguous counties), models were fitted similarly with the following predictors for NCORP and NCI site availability: first models, SVI overall theme continuous; second models, SVI overall theme dichotomized; third models, all 4 SVI themes and RUCC. Analyses were performed, and maps were created using SAS software, version 9.4 (SAS Institute, Cary, NC, USA). Geocoding was performed using Esri ArcGIS Pro software, version 2.5.0.

## Results

### County-level characteristics

**Figure 1.**
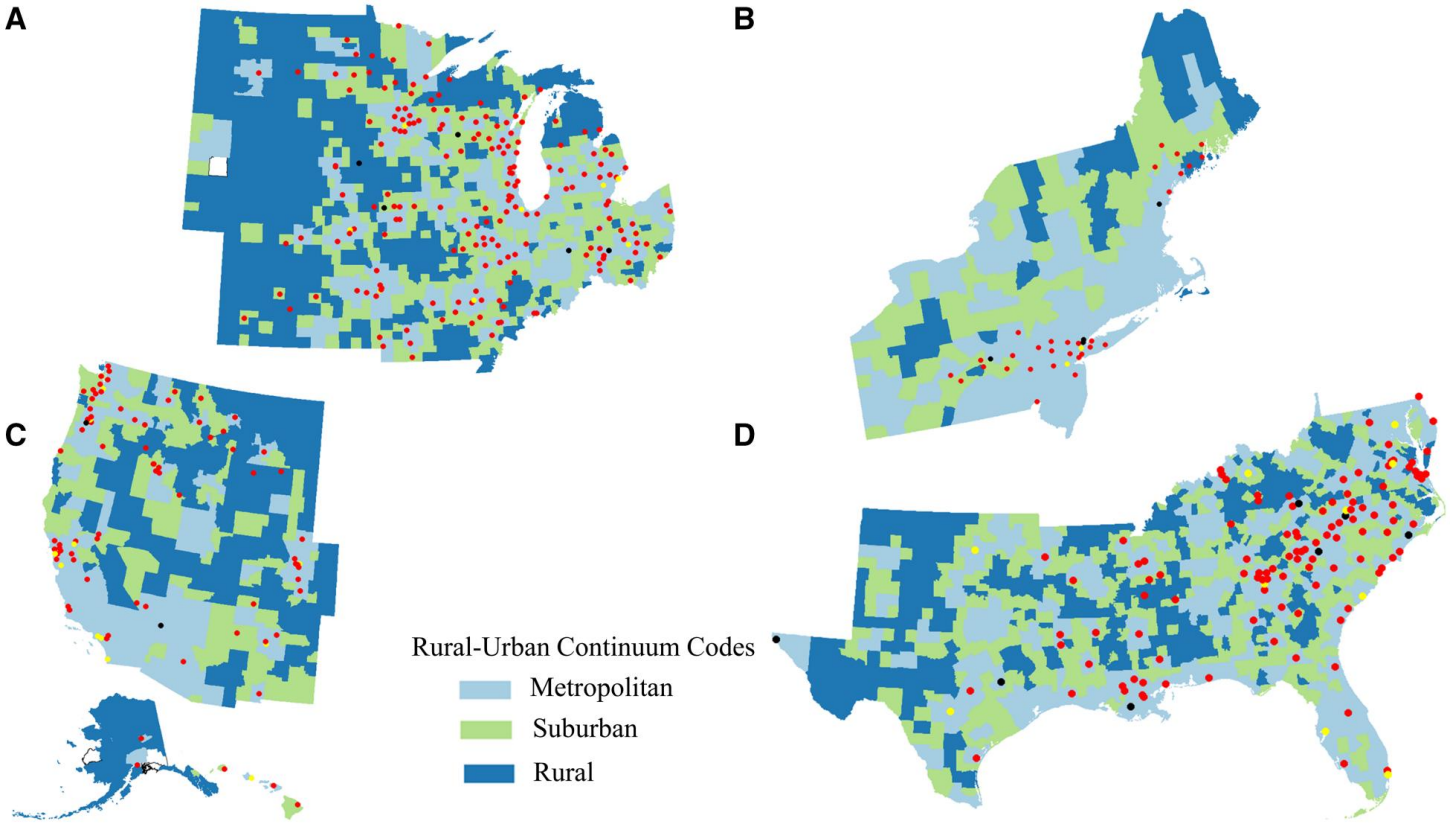
Map of the US counties by Rural-Urban Continuum Codes (metropolitan, suburban, rural) with **red dots** representing counties containing National Cancer Institute National Cancer Oncology Research Program sites, **black dots** representing counties containing National Cancer Institute sites, and **yellow dots** representing counties with both. **A)** Midwestern region. **B)** Northeastern region. **C)** Western region. **D)** Southern region.

**Figure 2.**
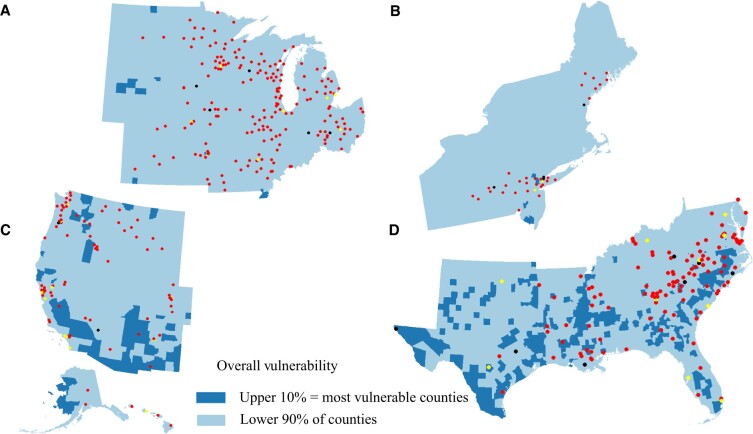
Map of the US counties by Social Vulnerability Index’s overall vulnerability theme of most vulnerable counties (upper 10%) and least vulnerable counties (lower 90%) with **red dots** representing counties containing National Cancer Institute National Cancer Oncology Research Program sites, **black dots** representing counties containing National Cancer Institute sites, and **yellow dots** representing counties with both. **A)** Midwestern region. **B)** Northeastern region. **C)** Western region. **D)** Southern region.

Of 3143 US counties, 3141 counties were included in our analysis. Two counties were excluded because of missing information on rurality (28) (Supplementary Table 1, available online). A total of 448 (14%) counties contained at least 1 NCORP site, and 53 (2%) counties had at least 1 NCI site (Table 1). Of the counties containing an NCORP site, 44% were in the Midwestern region and 71% were in metropolitan counties. All but 1 county with an NCI site were considered metropolitan. Furthermore, 32 metropolitan counties in the United States had both an NCORP and an NCI site (Figure 1). In all regions of the United States, NCORP sites were clustered in counties considered least vulnerable (Figure 2).

**Table 1.** County-level Social Vulnerability Index characteristics of total counties, counties without an NCORP or NCI sites, counties containing an NCORP site, counties containing an NCI site, and counties containing both NCORP and NCI sites^a^

Variables	Total	Counties without an NCORP or NCI site	Counties containing an NCORP site	Counties containing an NCI site	Counties containing both NCORP and NCI sites
	(n = 3141)	(n = 2672)	(n = 448)	(n = 53)	(n = 32)
Region, No. (%)					
Midwest	1055 (33.6)	85 (31.9)	197 (44.0)	13 (24.5)	8 (25.0)
Northeast	217 (6.9)	175 (6.6)	36 (8.0)	9 (17.0)	3 (9.4)
South	1422 (45.3)	1288 (48.2)	126 (28.1)	18 (34.0)	20 (31.2)
West	447 (14.2)	356 (13.3)	89 (19.9)	13 (24.5)	11 (34.4)
Rural-Urban Continuum Codes No. (%)					
1-3: Metropolitan	1166 (37.1)	828 (31.0)	318 (71.0)	52 (98.1)	32 (100)
4-6: Suburban	899 (28.6)	799 (29.9)	99 (22.1)	1 (1.9)	
7-9: Rural	1076 (34.3)	1045 (39.1)	31 (6.9)		
Overall theme, mean (SD)	0.50 (0.29)	0.50 (0.29)	0.51 (0.27)	0.67 (0.21)	0.70 (0.18)
Overall theme dichotomized No. (%)					
Most vulnerable, upper 10%	315 (10.0)	279 (10.4)	32 (7.1)	8 (15.1)	4 (12.5)
90% other counties	2826 (90.0)	2393 (89.6)	416 (92.9)	45 (84.9)	28 (87.5)
Socioeconomic status theme, mean (SD)	0.50 (0.29)	0.50 (0.29)	0.49 (0.27)	0.61 (0.26)	0.63 (0.24)
Percentage of persons below 150% poverty, mean (SD)	24.5 (8.5)	25.0 (8.6)	21.3 (7.0)	22.6 (5.8)	22.3 (6.2)
Unemployment rate estimate, mean (SD)	5.2 (2.6)	5.2 (2.7)	5.1 (1.6)	5.4 (1.5)	5.6 (1.4)
Percentage of housing cost burden, mean (SD)	22.3 (5.3)	21.7 (5.1)	25.5 (4.9)	30.3 (4.9)	30.7 (5.5)
Percentage of person with no high school diploma, mean (SD)	12.4 (6.0)	12.8 (6.2)	9.9 (4.3)	11.1 (4.5)	11.8 (4.6)
Percentage uninsured, mean (SD)	9.5 (5.1)	9.8 (5.3)	7.7 (3.4)	8.4 (4.3)	8.5 (3.9)
Household characteristics theme, mean (SD)	0.50 (0.29)	0.51 (0.29)	0.45 (0.27)	0.42 (0.29)	0.42 (0.29)
Percentage of persons aged 65 years and older, mean (SD)	19.2 (4.8)	19.6 (4.8)	17.4 (4.1)	14.3 (2.3)	14.1 (1.6)
Percentage of persons aged 17 years and younger, mean (SD)	22.1 (3.6)	22.1 (3.7)	22.1 (2.7)	21.4 (3.2)	21.6 (3.2)
Percentage of civilians with a disability, mean (SD)	16.0 (4.5)	16.4 (4.5)	13.7 (3.5)	11.7 (2.4)	11.6 (2.1)
Percentage of single-parent households, mean (SD)	5.9 (2.4)	5.8 (2.5)	6.2 (1.8)	6.8 (1.9)	6.8 (2.1)
Percentage of individuals who speak English less than well, mean (SD)	1.6 (2.7)	1.5 (2.7)	2.1 (2.7)	5.1 (4.0)	5.9 (4.31)
Racial and ethnic minority status theme, mean (SD)	0.50 (0.29)	0.48 (0.29)	0.57(0.26)	0.83 (0.14)	0.87 (0.10)
Percentage of individuals who are of racial and ethnic marginalized groups, mean (SD)	24.2 (20.2)	23.5 (20.2)	28.1 (19.9)	49.8 (18.5)	55.0 (16.5)
Housing type and transportation theme, mean (SD)	0.50 (0.29)	0.49 (0.29)	0.57 (0.27)	0.78 (0.13)	0.80 (0.12)
Percentage of multi-unit structures, mean (SD)	4.8 (5.8)	3.8 (4.6)	9.9 (8.5)	22.2 (13.9)	24.9 (16.7)
Percentage of mobile homes, mean (SD)	12.6 (9.5)	13.7 (9.6)	6.6 (6.2)	2.2 (2.2)	2.0 (1.9)
Percentage of overcrowded housing, mean (SD)	2.4 (2.4)	2.4 (2.5)	2.4 (1.9)	3.7 (2.6)	4.5 (2.9)
Percentage of household with no vehicles, mean (SD)	6.2 (4.5)	6.0 (4.2)	6.9 (5.9)	13.0 (13.4)	14.1 (16.0)
Percentage of individuals in group quarters, mean (SD)	3.5 (4.5)	3.6 (4.8)	2.8 (2.2)	2.9 (1.7)	2.5 (1.2)
Percentage without computer with broadband internet, mean (SD)	16.9 (7.6)	17.7 (7.7)	12.3 (5.1)	12.3 (5.1)	10.8 (3.9)

a All themes are percentile ranked from 0 to 1, with higher scores representing more vulnerability. Effect sizes can be found in Supplementary Table 2 (available online). NCI = National Cancer Institute; NCORP = National Cancer Institute Community Oncology Research Program; SD = standard deviation.

### Descriptive differences in counties by availability of NCORP and NCI sites

Compared with counties without an NCORP or NCI site, counties with an NCORP site had fewer individuals living in poverty (mean percentage = 21 vs 25, Cohen’s *d* = 0.44), without a high school diploma (mean percentage = 10 vs 13, Cohen’s *d* = 0.49), aged 65 year and older (mean percentage = 17 vs 20, Cohen’s *d* = 0.45), without internet (mean percentage = 12 vs 18, Cohen’s *d* = 0.74), and more multi-unit structures (mean percentage = 10 vs 4, Cohen’s *d* = 1.09). Compared with counties with neither site, counties with NCI sites were more racially diverse (mean percentage = 50 vs 24, Cohen’s *d* = 1.30) and had more multi-unit structures (mean percentage = 22 vs 4, Cohen’s *d* = 3.30). Counties with both an NCI and NCORP site were found to be the most vulnerable (overall SVI theme mean score = 0.70 [SD 0.18]) compared with counties without either site (overall SVI theme mean score = 0.50 [SD 0.29], Cohen’s *d* = 0.71), counties with an NCORP site (overall SVI theme mean score = 0.51 [0.27]), or counties with an NCI site (overall SVI theme mean score = 0.67 [0.21]). When assessing the county and its contiguous counties as having an NCORP (n = 1409, 45% of all counties) or NCI site (n = 263, 8%), these areas were less vulnerable when compared with counties with neither (Table 2). Supplementary Table 2 (available online) contains effect sizes for comparison groups.

**Table 2.** County-level Social Vulnerability Index characteristics of the counties containing a NCORP site and the contiguous counties and counties containing an NCI site and their contiguous counties^a^

Variables	Contiguous counties with an NCORP site	Contiguous counties with an NCI site
	(n = 1409)	(n = 263)
Region, No. (%)		
Midwest	585 (41.5)	74 (28.1)
Northeast	83 (5.9)	43 (16.4)
South	490 (34.8)	86 (32.7)
West	251 (17.8)	60 (22.8)
Rural-Urban Continuum Codes, No. (%)		
1-3: Metropolitan	682 (48.4)	216 (82.1)
4-6: Suburban	417 (29.6)	37 (14.1)
7-9: Rural	310 (22.0)	10 (3.8)
Overall theme, mean (SD)	0.47 (0.29)	0.45 (0.28)
Overall theme dichotomized		
Most vulnerable (upper 10%)	107 (7.6)	15 (5.7)
90% other counties	1302 (92.4)	248 (94.3)
Socioeconomic status theme, mean (SD)	0.47 (0.29)	0.43 (0.27)
Percentage of persons below 150% poverty, mean (SD)	22.8 (7.8)	18.0 (6.5)
Unemployment rate estimate, mean (SD)	5.2 (2.1)	4.9 (1.7)
Percentage of housing cost burden, mean (SD)	23.0 (5.0)	24.3 (5.1)
Percentage of person with no high school diploma, mean (SD)	11.3 (5.3)	10.1 (4.9)
Percentage uninsured, mean (SD)	8.4 (4.1)	7.4 (3.6)
Household characteristics theme, mean (SD)	0.47 (0.28)	0.43 (0.26)
Percentage of persons aged 65 years and older, mean (SD)	19.0 (4.6)	16.5 (3.7)
Percentage of persons aged 17 years and younger, mean (SD)	21.8 (3.2)	22.7 (3.1)
Percentage of civilians with a disability, mean (SD)	15.2 (4.0)	12.4 (3.2)
Percentage of single-parent households, mean (SD)	5.8 (2.1)	6.0 (1.6)
Percentage of individuals who speak English less than well, mean (SD)	1.6 (2.5)	3.0 (3.3)
Racial and ethnic minority status theme, mean (SD)	0.50 (0.28)	0.61 (0.26)
Percentage of individuals who are of racial and ethnic marginalized groups, mean (SD)	23.6 (19.2)	31.0 (21.0)
Housing type and transportation theme, mean (SD)	0.48 (0.29)	0.47 (0.28)
Percentage of multi-unit structures, mean (SD)	5.6 (6.6)	9.89 (10.2)
Percentage of mobile homes, mean (SD)	11.34 (9.5)	7.1 (7.9)
Percentage of overcrowded housing, mean (SD)	2.3 (1.8)	2.8 (2.3)
Percentage of household with no vehicles, mean (SD)	5.9 (4.2)	6.0 (7.1)
Percentage of individuals in group quarters, mean (SD)	3.2 (3.9)	2.3 (1.9)
Percentage without computer with broadband internet, mean (SD)	15.3 (6.9)	10.7 (4.8)

a All themes are percentile ranked from 0 to 1, with higher scores representing more vulnerability. NCI = National Cancer Institute; NCORP = National Cancer Institute Community Oncology Research Program; SD = standard deviation.

### Internet access results

Compared with counties without an NCORP or NCI site, counties with an NCORP and NCI site had fewer individuals without internet (mean percentages = 12 vs 18, Cohen’s *d* = 0.74; Table 1).

### Model results of singular counties

When assessing the singular county results, the most vs least vulnerable counties (overall SVI theme) had 31% lower likelihood of containing an NCORP site (95% CI = 0.49 to 0.97; Table 3). However, NCI sites were more likely to be in more vulnerable counties; the most vs least vulnerable counties had 1.59 times the likelihood of NCI site availability (95% CI = 0.76 to 3.35). In the model assessing the 4 SVI themes and RUCC for NCORP availability, all were statistically significant. With 1 standard deviation of socioeconomic and household theme scores for counties, there was a 24% (95% CI = 0.67 to 0.87) and 11% (95% CI = 0.80 to 0.99) lower likelihood of NCORP site availability, respectively. Conversely, for a standard deviation increase in the racial and ethnicity and housing type and transportation theme score, NCORP availability was 22% (95% CI = 1.10 to 1.36) and 33% (95% CI = 1.20 to 1.47) more likely, respectively. Furthermore, compared with rural areas, metropolitan and suburban areas had higher likelihood of an available NCORP site (RR = 8.35, 95% CI = 5.85 to 11.91; RR = 3.76, 95% CI = 2.52 to 5.61, respectively). Results were similar, but of a larger magnitude, for the models assessing NCI availability. Notably, with 1 standard deviation increase in the racial and ethnicity theme score for singular counties, NCI availability was 6 times more likely (RR = 6.00, 95% CI = 4.09 to 8.81).

**Table 3.** Model-estimated risks ratios, 95% confidence intervals, and predicted proportions evaluating the association between county-level characteristics and availability of NCORP and NCI sites in singular counties (n= 3141)^a^

	Risk ratio	Predicted proportion
	(95% CI)	
**Availability of NCORP sites**		
Model 1 (n = 3141), reference proportion 0.14		
Overall theme continuous, SD increase	1.05 (0.97 to 1.14)	0.15
Model 2 (n = 3141)		
Overall theme dichotomized		
Upper 10%, most vulnerable	**0.69 (0.49 to 0.97)**	0.10
Other 90% of counties, least vulnerable	Referent	0.15
Model 3 (n = 3141), reference proportion 0.09		
Socioeconomic status theme, SD increase	**0.76 (0.67 to 0.87)**	0.07
Household characteristics theme, SD increase	**0.89 (0.80 to 0.99)**	0.08
Racial and ethnic minority status theme, SD increase	**1.22 (1.10 to 1.36)**	0.11
Housing type and transportation theme, SD increase	**1.33 (1.20 to 1.47)**	0.12
Rural-Urban Continuum Codes		
Metropolitan	**8.35 (5.85 to 11.91)**	0.24
Suburban	**3.76 (2.52 to 5.61)**	0.11
Rural	Referent	0.03
**Availability of NCI sites**		
Model 1 (n = 3141), reference proportion 0.01		
Overall theme continuous, SD increase	**1.91 (1.50 to 2.45)**	0.03
Model 2 (n = 3141)		
Overall theme dichotomized		
Upper 10%, most vulnerable	1.59 (0.76 to 3.35)	0.03
Other 90% of counties, least vulnerable	Referent	0.02
Models 3 (n = 3141), reference proportion 0.004		
Socioeconomic status theme, SD increase	**0.71 (0.53 to 0.96)**	0.003
Household characteristics theme, SD increase	**0.51 (0.40 to 0.65)**	0.002
Racial and ethnic minority status theme, SD increase	**6.00 (4.09 to 8.81)**	0.02
Housing type and transportation theme, SD increase	**2.04 (1.58 to 2.63)**	0.007

a Models 1 and 2 only contain the Social Vulnerability Index overall theme; model 3 contains all 4 Social Vulnerability Index themes (NCI only) and Rural-Urban Continuum Codes. All themes are percentile ranked from 0 to 1, with higher scores representing more vulnerability. **Bolded values** represent statistical significance at the 0.05 alpha level. CI = confidence interval; NCI = National Cancer Institute; NCORP = National Cancer Institute Community Oncology Research Program; SD = standard deviation.

### Model results of with singular and contiguous counties

When assessing the likelihood of counties and their corresponding contiguous counties having at least 1 site, NCORP results were similar to the NCORP singular county models except with increases in racial and ethnicity theme score for counties and their contiguous counties, we found there was no difference in availability of NCORP sites (RR = 1.03, 95% CI = 0.98 to 1.08; Table 4). For models assessing availability of an NCI site in counties and contiguous counties, SVI overall theme results were similar to the NCORP model results; when overall vulnerability increased, there was a 17% lower likelihood of having NCI site availability (overall theme continuous: RR = 0.83, 95% CI = 0.75 to 0.94), which is opposite of the singular county NCI model. Furthermore, for the model containing all 4 themes and RUCC, results for NCI availability were similar to the singular NCORP model expect for the racial and ethnic minority status theme. With 1 standard deviation increase in the racial and ethnicity theme score, there is 1.97 times the likelihood of an available NCI site (95% CI 1.68 to 2.32). For both the NCORP and NCI models, metropolitan and suburban areas had higher likelihoods of having NCORP or NCI sites when compared with rural areas.

**Table 4.** Model-estimated risks ratios, 95% confidence intervals, and predicted proportions evaluating the association between county-level characteristics and availability of NCORP and NCI sites in contiguous counties (n = 3141)^a^

	Risk ratio	Predicted proportion
	(95% CI)	
**Availability of NCORP sites**		
Model 1 (n = 3141), reference proportion 0.45		
Overall theme continuous, SD increase	**0.91 (0.87 to 0.94)**	0.41
Model 2 (n = 3141)		
Overall theme dichotomized		
Upper 10%, most vulnerable	**0.74 (0.63 to 0.86)**	0.34
Other 90% of counties, least vulnerable	Referent	0.46
Model 3 (n = 3141), reference proportion 0.43		
Socioeconomic status theme, SD increase	**0.92 (0.86 to 0.98)**	0.39
Household characteristics theme, SD increase	0.95 (0.90 to 1.00)	0.40
Racial and ethnic minority status theme, SD increase	1.03 (0.98 to 1.08)	0.44
Housing type and transportation theme, SD increase	0.96 (0.92 to 1.01)	0.41
Rural-Urban Continuum Codes		
Metropolitan	**2.03 (1.82 to 2.26)**	0.57
Suburban	**1.72 (1.53 to 1.93)**	0.48
Rural	Referent	0.28
**Availability of NCI sites**		
Model 1 (n = 3141), reference proportion 0.08		
Overall theme continuous, SD increase	**0.83 (0.75 to 0.94)**	0.07
Model 2 (n = 3141)		
Overall theme dichotomized		
Upper 10%, most vulnerable	**0.54 (0.33 to 0.90)**	0.05
Other 90% of counties, least vulnerable	Referent	0.09
Model 3 (n = 3141), reference proportion 0.04		
Socioeconomic status theme, SD increase	**0.59 (0.49 to 0.72)**	0.02
Household characteristics theme, SD increase	0.92 (0.79 to 1.06)	0.03
Racial and ethnic minority status theme, SD increase	**1.97 (1.68 to 2.32)**	0.07
Housing type and transportation theme, SD increase	0.89 (0.78 to 1.01)	0.03
Rural-Urban Continuum Codes		
Metropolitan	**16.20 (8.64 to 30.39)**	0.13
Suburban	**5.41 (2.68 to 10.91)**	0.04
Rural	Referent	0.01

a Models 1 and 2 only contain the Social Vulnerability Index overall theme; model 3 contains all 4 Social Vulnerability Index themes and Rural-Urban Continuum Codes. All themes are percentile ranked from 0 to 1, with higher scores representing more vulnerability. **Bolded values** represent statistical significance at the 0.05 alpha level. CI=confidence interval; NCI=National Cancer Institute; NCORP = National Cancer Institute Community Oncology Research Program; SD = standard deviation.

## Discussion

This study found that the majority of US counties does not have access to an NCORP site, a federally funded program designed to bring cancer clinical trials to the community via the network’s resources (ie, financial, staffing, medical equipment). This finding is important because it highlights systematic area-level factors that could influence clinical trial access and, thus, representation. Singular counties with higher proportions of marginalized racial and ethnic groups have higher likelihoods of having access to NCORP sites, which is likely explained by NCORP’s Minority/Underserved Community Sites. These sites—which are categorized as Minority/Underserved Sites as they serve at least 30% racial and ethnic marginalized groups or rural residents (29)—appear to be appropriately located in singular counties that serve racially and ethnically diverse populations. However, for contiguous counties, as marginalized racial groups increased, there was a similar likelihood of NCORP site availability, while conversely, there was a higher likelihood of NCI site availability. Previous studies have assessed availability of specific clinical trials within specific cancer types at the county level, such as Wang and colleagues (30), who found that US counties with higher proportions of African Americans are less likely to have access to any prostate cancer clinical trials. Additionally, Grant and colleagues (31) assessed the association between SVI themes and availability of multiple myeloma trials within North Carolina and found similar results to Wang et al. (30). Although these studies assessed clinical trial availability differently than ours, the need for more NCORP Community Sites to serve a wider catchment area of marginalized racial groups who are underrepresented in clinical trials is evident throughout. Additionally, increasing accessibility is a necessary piece in overcoming underrepresentation of marginalized racial groups, especially as NCORP sites have reported the population they serve is predominantly White (9). There is currently no other sociodemographic information available on NCORP sites’ patient population.

Another interesting finding is that all NCI sites were located in metropolitan counties. Additionally, metropolitan and suburban vs rural counties had higher likelihoods of having availability of NCORP sites. However, patients outside metropolitan areas may have distinct risks and health-care needs. Research has shown that the health of the community and the built environment is influential on individual-level health (21,32-36). Zhang and colleagues (37) found that there is a strong relationship between the proximity to landfills and a diagnosis of bladder, breast, and liver cancer. Furthermore, they found the proximity to major roads and industry are associated with lung cancer. Particularly, more urban communities can drive greater access to health care as, often, urban areas have more public transportation, whereas rural areas face distance barriers (38-40).

Compounded with rurality, our study found that as the socioeconomic theme increased (ie, increasing vulnerability) for contiguous counties, there was 8% and 41% lower likelihood of availability of NCORP and NCI sites, respectively. The socioeconomic status (SES) of the community is important, as higher levels of SES have been shown to be associated with more access to health care (41,42) and clinical trial enrollment (43). One’s SES status is associated with not only place, race, and insurance status but also education, income, and employment, which affect cancer outcomes (44). For example, individuals living in rural or lower SES locations often present to clinic in more advanced cancer stages, which is associated with poorer survival, and they more often receive delayed cancer care (45-47). These results point to the issue many programs face—we may not be reaching the truly vulnerable patients. There is a need to include additional NCORP sites for patients who reside in rural or poorer areas because Community Sites are increasingly closing or being acquired by other hospitals (48). This would ensure increased access to clinical trials for patients residing in vulnerable areas.

Additionally, the inclusion of the individuals without broadband internet is of great importance for the future of clinical trials. Since the COVID-19 pandemic, there has been an increase in the use of telemedicine; furthermore, clinical trials are including more use of telemedicine in hopes of alleviating transportation barriers and increasing participation (49-51). The Pew Research Center has found that those with increasing education and income more often have in-home internet (52). Because individuals in counties with these sites more often have internet than counties without, this should be a consideration when expanding NCORP sites. If clinical trials are to increase the use of telemedicine, sites will want to mitigate inequities surrounding internet access, especially as those who do not have internet may already be likely to be underrepresented and, thus, more likely to have poorer outcomes (eg, disability, disease, death) (53). Future research is needed to understand the intersectionality of transportation obstacles, financial barriers, and internet access, especially as it relates to health care seeking behaviors and clinical trial participation.

This study should be considered in light of several limitations. As this is an exploratory analysis, we were unable to ascertain a causal link between county-level data and inclusion of NCORP or NCI sites. Consideration of when sites were included in NCORP or designated NCI cancer centers is outside the scope of this research. In addition, we were unable to determine how the counties with access to multiple NCORP and/or NCI sites differ from counties with 1 site and if there is a “dose” effect. Additionally, by using exploratory associations at the county level, there is potential that ecological fallacies are present. Our assumption that patients are willing and able to travel to a different county is also a limitation to this analysis, and further work is needed to understand each hospital’s catchment area. Additionally, our study is limited in the availability of internet access data at the county level. Finally, these findings are meant to be interpreted at the county level and therefore may not represent individual-level data and experiences. Of note, strengths of this study are the robust use of nationwide address and census data, teasing out of NCORP and NCI sites, and the use of domains of vulnerability.

This study found NCORP sites are less often located in counties and their contiguous counties with populations underrepresented in clinical trials. Conversely, NCI sites are more often located in areas that serve marginalized racial and ethnic groups. There are fewer NCI sites than NCORP sites; therefore, the reach of NCORP is much greater as is the need, especially in poorer areas, as these individuals face the greatest disease burden. Additionally, counties without access to NCORP or NCI sites have less access to internet. More research is needed to understand how clinical trial representation will increase if NCORP sites strategically collaborate with hospitals in socially vulnerable areas.

## Supplementary Material

pkae038_Supplementary_Data

## Data Availability

Data derived from sources in the public domain (National Cancer Institute: https://gis.cancer.gov/ncicatchment/; NCI Community Oncology Research Program: https://ncorp.cancer.gov/findasite/; Centers for Disease Control and Prevention/Agency for Toxic Substances and Disease Registry’s Social Vulnerability Index: https://www.atsdr.cdc.gov/placeandhealth/svi/index.html; Health Resources and Services Administration’s Area Health Resource Files: https://data.hrsa.gov/topics/health-workforce/ahrf).
