# Supplementary material for: Why location matters: associations between county-level characteristics and availability of National Cancer Oncology Research Program and National Cancer Institute sites
Source: JNCI Cancer Spectr. 2024 May 14;8(3):pkae038. doi: 10.1093/jncics/pkae038 (PMC11163183; doi:10.1093/jncics/pkae038)
Supplement: pkae038_Supplementary_Data [file pkae038_supplementary_data.docx]

Supplementary Material

Supplementary Table 1. County-level Social Vulnerability Index characteristics of counties included in the analysis (n=3141) and excluded (n=2).

|  | **Included in the analysis** | **Excluded from the analysis** | **Cramer’s V or Cohen’s d** |
| --- | --- | --- | --- |
|  | **N=3141** | **n=2** |  |
| **Region** |  |  | 0.06 |
| Midwest | 1055 (33.6) | -- |  |
| Northeast | 217 (6.9) | -- |  |
| South | 1422 (45.3) | -- |  |
| West | 447 (14.2) | 2 (100) |  |
| **Rural-Urban Continuum Codes** |  |  | NA |
| 1-3: Metropolitan | 1166 (37.1) | -- |  |
| 4-6: Suburban | 899 (28.6) | -- |  |
| 7-9: Rural | 1076 (34.3) | -- |  |
| **Overall theme, mean (SD)** | 0.50 (0.29) | 0.31 (0.11) | 0.67 |
| **Overall theme dichotomized** |  |  | 0.01 |
| Most vulnerable (upper 10%) | 315 (10.0) | -- |  |
| 90% other counties | 2826 (90.0) | 2 (100) |  |
| **Socioeconomic status theme, mean (SD)** | 0.50 (0.29) | 0.20 (0.16) | 1.04 |
| Percentage of persons below 150% poverty, mean (SD) | 24.5 (8.5) | 13.4 (9.3) | 1.30 |
| Unemployment rate estimate, mean (SD) | 5.2 (2.6) | 4.2 (2.0) | 0.39 |
| Percentage of housing cost burden, mean (SD) | 22.3 (5.3) | 17.0 (0.6) | 1.00 |
| Percentage of person with no high school diploma, mean (SD) | 12.4 (6.0) | 4.5 (0.1) | 1.31 |
| Percentage uninsured, mean (SD) | 9.5 (5.1) | 17.4 (3.5) | 1.54 |
| **Household characteristics theme, mean (SD)** | 0.50 (0.29) | 0.18 (0.23) | 1.12 |
| Percentage of persons aged 65 & older, mean (SD) | 19.2 (4.8) | 12.9 (0.8) | 1.33 |
| Percentage of persons aged 17 & younger, mean (SD) | 22.1 (3.6) | 24.6 (4.5) | 0.70 |
| Percentage of civilians with a disability, mean (SD) | 16.0 (4.5) | 13.8 (3.6) | 0.50 |
| Percentage of single-parent households, mean (SD) | 5.9 (2.4) | 4.8 (2.6) | 0.47 |
| Percentage of individuals who speak English “less than well”, mean (SD) | 1.6 (2.7) | 0.3 (0.4) | 0.49 |
| **Racial & ethnic minority status theme, mean (SD)** | 0.50 (0.29) | 0.73 (0.13) | 0.81 |
| Percentage of individuals who are of racial & ethnic marginalized groups, mean (SD) | 24.2 (20.2) | 35.0 (11.7) | 0.53 |
| **Housing type & transportation theme, mean (SD)** | 0.50 (0.29) | 0.65 (0.39) | 0.52 |
| Percentage of multi-unit structures, mean (SD) | 4.8 (5.8) | 4.0 (4.2) | 0.14 |
| Percentage of mobile homes, mean (SD) | 12.6 (9.5) | 12.1 (7.4) | 0.06 |
| Percentage of overcrowded housing, mean (SD) | 2.4 (2.4) | 4.6 (0.4) | 0.92 |
| Percentage of household with no vehicles, mean (SD) | 6.2 (4.5) | 5.9 (2.3) | 0.07 |
| Percentage of individuals in group quarters, mean (SD) | 3.5 (4.5) | 2.2 (1.9) | 0.30 |
| **Percentage without computer that has broadband internet, mean (SD)** | 16.9 (7.6) | 14.7 (5.3) | 0.30 |

NCORP= National Cancer Institute Community Oncology Research Program; NCI=National Cancer Institute; SD=standard deviation. No value given due to missing data.

Supplementary Table 2. County-level Social Vulnerability Index characteristics of total counties (N=3141), counties without an NCORP nor NCI sites (n=2672), counties containing an NCORP site (n=448), counties containing an NCI site (n=53), and counties containing both NCORP and NCI sites (n=32).

|  | **Total** | **Counties without an NCORP nor NCI site** | **Counties containing an NCORP site** | **Effect sizes: counties with vs without an NCORP site** | **Counties containing an NCI site** | **Effect sizes: counties with vs without an NCI site** | **Counties containing both NCORP and NCI sites** | **Effect sizes: counties with both sites vs all others** |
| --- | --- | --- | --- | --- | --- | --- | --- | --- |
|  | **N=3141** | **n=2672** | **n=448** |  | **n=53** |  | **n=32** |  |
|  | n (%) or mean (SD) | | |  | n (%) or mean (SD) |  | n (%) or mean (SD) |  |
| **Region** |  |  |  | 0.14 |  | 0.07 |  | 0.06 |
| Midwest | 1055 (33.6) | 85 (31.9) | 197 (44.0) |  | 13 (24.5) |  | 8 (25.0) |  |
| Northeast | 217 (6.9) | 175 (6.6) | 36 (8.0) |  | 9 (17.0) |  | 3 (9.4) |  |
| South | 1422 (45.3) | 1288 (48.2) | 126 (28.1) |  | 18 (34.0) |  | 20 (31.2) |  |
| West | 447 (14.2) | 356 (13.3) | 89 (19.9) |  | 13 (24.5) |  | 11 (34.4) |  |
| **Rural-Urban Continuum Codes** |  |  |  | 0.3 |  | 0.17 |  | 0.13 |
| 1-3: Metropolitan | 1166 (37.1) | 828 (31.0) | 318 (71.0) |  | 52 (98.1) |  | 32 (100) |  |
| 4-6: Suburban | 899 (28.6) | 799 (29.9) | 99 (22.1) |  | 1 (1.9) |  |  |  |
| 7-9: Rural | 1076 (34.3) | 1045 (39.1) | 31 (6.9) |  |  |  |  |  |
| **Overall theme, mean (SD)** | 0.50 (0.29) | 0.50 (0.29) | 0.51 (0.27) | 0.06 | 0.67 (0.21) | 0.61 | 0.70 (0.18) | 0.71 |
| **Overall theme dichotomized** |  |  |  | 0.04 |  | 0.02 |  | 0.01 |
| Most vulnerable (upper 10%) | 315 (10.0) | 279 (10.4) | 32 (7.1) |  | 8 (15.1) |  | 4 (12.5) |  |
| 90% other counties | 2826 (90.0) | 2393 (89.6) | 416 (92.9) |  | 45 (84.9) |  | 28 (87.5) |  |
| **Socioeconomic status theme, mean (SD)** | 0.50 (0.29) | 0.50 (0.29) | 0.49 (0.27) | 0.05 | 0.61 (0.26) | 0.38 | 0.63 (0.24) | 0.46 |
| Percentage of persons below 150% poverty, mean (SD) | 24.5 (8.5) | 25.0 (8.6) | 21.3 (7.0) | 0.44 | 22.6 (5.8) | 0.23 | 22.3 (6.2) | 0.26 |
| Unemployment rate estimate, mean (SD) | 5.2 (2.6) | 5.2 (2.7) | 5.1 (1.6) | 0.06 | 5.4 (1.5) | 0.09 | 5.6 (1.4) | 0.14 |
| Percentage of housing cost burden, mean (SD) | 22.3 (5.3) | 21.7 (5.1) | 25.5 (4.9) | 0.73 | 30.3 (4.9) | 1.57 | 30.7 (5.5) | 1.63 |
| Percentage of person with no high school diploma, mean (SD) | 12.4 (6.0) | 12.8 (6.2) | 9.9 (4.3) | 0.49 | 11.1 (4.5) | 0.22 | 11.8 (4.6) | 0.1 |
| Percentage uninsured, mean (SD) | 9.5 (5.1) | 9.8 (5.3) | 7.7 (3.4) | 0.41 | 8.4 (4.3) | 0.23 | 8.5 (3.9) | 0.21 |
| **Household characteristics theme, mean (SD)** | 0.50 (0.29) | 0.51 (0.29) | 0.45 (0.27) | 0.21 | 0.42 (0.29) | 0.3 | 0.42 (0.29) | 0.29 |
| Percentage of persons aged 65 & older, mean (SD) | 19.2 (4.8) | 19.6 (4.8) | 17.4 (4.1) | 0.45 | 14.3 (2.3) | 1.06 | 14.1 (1.6) | 1.09 |
| Percentage of persons aged 17 & younger, mean (SD) | 22.1 (3.6) | 22.1 (3.7) | 22.1 (2.7) | 0.01 | 21.4 (3.2) | 0.19 | 21.6 (3.2) | 0.14 |
| Percentage of civilians with a disability, mean (SD) | 16.0 (4.5) | 16.4 (4.5) | 13.7 (3.5) | 0.6 | 11.7 (2.4) | 0.99 | 11.6 (2.1) | 0.99 |
| Percentage of single-parent households, mean (SD) | 5.9 (2.4) | 5.8 (2.5) | 6.2 (1.8) | 0.18 | 6.8 (1.9) | 0.4 | 6.8 (2.1) | 0.38 |
| Percentage of individuals who speak English “less than well”, mean (SD) | 1.6 (2.7) | 1.5 (2.7) | 2.1 (2.7) | 0.23 | 5.1 (4.0) | 1.33 | 5.9 (4.31) | 1.64 |
| **Racial & ethnic minority status theme, mean (SD)** | 0.50 (0.29) | 0.48 (0.29) | 0.57(0.26) | 0.3 | 0.83 (0.14) | 1.17 | 0.87 (0.10) | 1.32 |
| Percentage of individuals who are of racial & ethnic marginalized groups, mean (SD) | 24.2 (20.2) | 23.5 (20.2) | 28.1 (19.9) | 0.23 | 49.8 (18.5) | 1.3 | 55.0 (16.5) | 1.55 |
| **Housing type & transportation theme, mean (SD)** | 0.50 (0.29) | 0.49 (0.29) | 0.57 (0.27) | 0.27 | 0.78 (0.13) | 1 | 0.80 (0.12) | 1.06 |
| Percentage of multi-unit structures, mean (SD) | 4.8 (5.8) | 3.8 (4.6) | 9.9 (8.5) | 1.09 | 22.2 (13.9) | 3.3 | 24.9 (16.7) | 3.72 |
| Percentage of mobile homes, mean (SD) | 12.6 (9.5) | 13.7 (9.6) | 6.6 (6.2) | 0.75 | 2.2 (2.2) | 1.12 | 2.0 (1.9) | 1.12 |
| Percentage of overcrowded housing, mean (SD) | 2.4 (2.4) | 2.4 (2.5) | 2.4 (1.9) | 0.01 | 3.7 (2.6) | 0.58 | 4.5 (2.9) | 0.9 |
| Percentage of household with no vehicles, mean (SD) | 6.2 (4.5) | 6.0 (4.2) | 6.9 (5.9) | 0.19 | 13.0 (13.4) | 1.57 | 14.1 (16.0) | 1.8 |
| Percentage of individuals in group quarters, mean (SD) | 3.5 (4.5) | 3.6 (4.8) | 2.8 (2.2) | 0.19 | 2.9 (1.7) | 0.14 | 2.5 (1.2) | 0.23 |
| **Percentage without computer with broadband internet, mean (SD)** | 16.9 (7.6) | 17.7 (7.7) | 12.3 (5.1) | 0.74 | 12.3 (5.1) | 0.81 | 10.8 (3.9) | 0.82 |
